# Application of machine learning models in the capacity prediction of RCFST columns

**DOI:** 10.1038/s41598-023-48044-1

**Published:** 2023-11-27

**Authors:** Khaled Megahed, Nabil Said Mahmoud, Saad Elden Mostafa Abd-Rabou

**Affiliations:** Department of Structural Engineering, Mansoura University, PO BOX 35516, Mansoura, Egypt

**Keywords:** Civil engineering, Mechanical properties, Scientific data, Statistics

## Abstract

Rectangular concrete-filled steel tubular (RCFST) columns are widely used in structural engineering due to their excellent load-carrying capacity and ductility. However, existing design equations often yield different design results for the same column properties, leading to uncertainty for engineering designers. Furthermore, basic regression analysis fails to precisely forecast the complicated relation between the column properties and its compressive strength. To overcome these challenges, this study suggests two machine learning (ML) models, including the Gaussian process (GPR) and the extreme gradient boosting model (XGBoost). These models employ a range of input variables, such as the geometric and material properties of RCFST columns, to estimate their strength. The models are trained and evaluated based on two datasets consisting of 958 axially loaded RCFST columns and 405 eccentrically loaded RCFST columns. In addition, a unitless output variable, termed the strength index, is introduced to enhance model performance. From evolution metrics, the GPR model emerged as the most accurate and reliable model, with nearly 99% of specimens with less than 20% error. In addition, the prediction results of ML models were compared with the predictions of two existing standard codes and different ML studies. The results indicated that the developed ML models achieved notable enhancement in prediction accuracy. In addition, the Shapley additive interpretation (SHAP) technique is employed for feature analysis. The feature analysis results reveal that the column length and load end-eccentricity parameters negatively impact compressive strength.

## Introduction

A concrete-filled steel tube (CFST) is a composite structural element composed of a steel tube and an inner concrete infill to optimize the usage of the two materials, resulting in favorable mechanical behavior over conventional reinforced concrete or pure steel elements. The confinement provided by the steel tube enhances the concrete capacity and ductility, while the infill concrete restrains the inner local buckling of the steel tube^1,2^. Therefore, CFST columns are highly employed for their exceptional strength and excellent performance, making them the most suitable choice in many construction applications, such as buildings and bridges.

Many experimental studies have been conducted to understand the axial behavior of CFST columns^3–5^. In the early loading stage of CFST columns, no significant interaction stress is created between the outer tube and the concrete infill^6^ as the lateral strain of steel material is higher than that of concrete at the beginning of loading. However, as the loading progresses, concrete volume rapidly increases at the elastic–plastic stage, reducing the separation and activating confining stresses. These confining stresses increase gradually until the peak load is reached and a large contacting pressure is formed^6^. The post-peak behavior mainly depends on the confinement provided by the outer tube^7^. It was noticed that increasing the thickness of the steel tube and using relatively low-strength concrete can enhance the ductility and post-peak performance of CFST columns^6^.

Compressive resistance stands as the primary mechanical characteristic of CFST columns. Due to the complex behavior of CFST columns, exploring various techniques to extract their compressive resistance can facilitate the comprehension of their behavior. The most commonly used techniques for predicting the compressive strength of CFST columns are experimental investigation and finite element analysis^8,9^. While experimental analysis yields valuable findings, it is both labor-intensive and costly. Furthermore, finite element analysis requires high computational resources, a comprehensive understanding of the complex behavior of concrete material under confinement, and appropriate modeling of the concrete-steel interface. Many design codes are available for predicting compressive strength CFST columns, including Eurocode 4^10^ and AISC 360-22^11^. However, it should be noted that they have specific application scopes and produce different results due to the restricted nonlinear mapping between the inputs and outputs, raising concerns about their prediction accuracy.

The machine learning (ML) technique can be employed as an alternative to predict the axial capacity of RCFST columns. ML has emerged as a promising tool to tackle complicated problems by conserving resources using existing experimental tests and lessening the necessity for additional testing^12–17^. Recently, many studies have employed various ML algorithms, including artificial neural networks (ANNs), support vector regression (SVR)^18^, and Gaussian process (GPR)^19^, to develop empirical formulas and statistical models for predicting the compressive strength of RCFST columns based on experimental tests collected from the literature and have provided positive satisfactory outcomes^20–26^.

For example, Ahmadi et al.^12,13^ employed ANN to predict the compressive resistance of short CFST columns and derived a design expression for axially loaded CFST columns. Du et al.^14^ utilized ANNs to forecast the ultimate capacity of stub rectangular concrete-filled steel tube (RCFST) columns using 305 specimens collected from the literature. Le et al.^15^ used ANNs to predict the axial load strength of square and rectangular CFST columns using a dataset of 880 specimens. Tran et al.^16^ used a database of 300 axially loaded experimental tests to compute the axial capacity of the squared CFT column using ANNs. Furthermore, Zarringol et al.^17^ utilized four separate databases for predicting the compressive resistance of circular and rectangular CFST columns under axial and eccentric loading and proposed empirical equations and strength reduction factors to facilitate practical design applications. Le^20^ proposed a GPR-based ML model for the ultimate strength of square CFST columns. In addition, Naser et al.^21^ employed a genetic algorithm (GA) and gene expression programming (GEP) for extracting the strength of rectangular and circular CFST columns using 3103 test results. Nguyen et al.^23,24^ proposed two ANN models trained with 99 concentrically loaded and 662 eccentrically loaded rectangular CFST specimens. Memarzadeh et al.^25^ predicted the axial capacity of square CFST specimens by training GEP and ANN models using 347 axially loaded rectangular CFST specimens. Wang et al.^26^ trained three models, including SVR, ANN, and random forest (RFR) models, to predict the strength of eccentrically loaded rectangular CFST specimens. Table 1 summarizes the recent machine learning models in predicting square and rectangular CFST column strength.

**Table 1:** Summary of recent machine learning models for predicting the strength of square and rectangular CFST columns.

Reference	Loading (number) Type [Split ratio%]	Models	Input (output)	Statistical criteria
Ren^22^	Concentric (180) Square [70:30]	SVR, PSO	*L, H, t, f*_*y*_*, f*_*c*_*’, E*_*c*_*, E*_*s*_ (*P*_*u*_)	(Train) R^2^ = 0.932, MAPE% = 14.3, MAE = 239, RMSE = 314 (Test) R^2^ = 0.914, MAPE% = 14.5, MAE = 227, RMSE = 304
Tran^16^	Concentric (300) Square [85:15]	ANN	*L, H, t, f*_*y*_*, f*_*c*_*’* (*P*_*u*_)	R^2^ = 0.99599, MSE = 0.011535
Nguyen^23^	Concentric (99) Rectangular [80:20]	ANN	*H, B, t, L, f*_*y*_*, f*_*c*_’ (*P*_*u*_)	R^2^ = 0.978, RMSE = 47.9. MAE = 49.2,
Zarringol^17^	Concentric (895) Eccentric (392) Rectangular [85:15]	ANN	*L,*$\sqrt{{B}^{2}+{H}^{2}}$*, t, f*_*y*_*, f*_*c*_’ (*P*_*u*_)	Concentric: R^2^ = 0.99, μ = 1.0, CoV = 0.138 Eccentric: R^2^ = 0.9959, μ = 1.0, CoV = 0.107
Le^20^	Concentric (314) Square [70:30]	GPR	*B, B/t, L/B, f*_*y*_*, f*_*c*_’ (*P*_*u*_)	R^2^ = 0.97, RMSE = 377.3kN, MAE = 257.8kN, MAPE = 17.55,
Nguyen^24^	Eccentric (662) Rectangular [80:20]	ANN	*H, B, t, L, f*_*y*_*, f*_*c*_’*, e* (*P*_*u*_)	R^2^ = 0.994, MSE = 0.506, RMSE = 0.225, MAPE% = 12.1
Naser^21^	Concentric (979) Eccentric (394) Rectangular [70:30]	GA, GEP	*L, H, B, t, f*_*y*_*, f*_*c*_’*, e*_*t*_*, e*_*b*_ (*P*_*u*_)	Concentric μ(GA) = 1.02, μ(GEP) = 1.06, CoV(GA) = 0.13, CoV(GEP) = 0.15 MAE(GA) = 202, MAE(GEP) = 238, RMSE(GA) = 295, RMSE(GEP) = 340 Eccentric μ(GA) = 1.26, μ(GEP) = 0.96, CoV(GA) = 0.18, CoV(GEP) = 0.20 MAE(GA) = 168, MAE(GEP) = 168, RMSE(GA) = 219, RMSE(GEP) = 251
Le^15^	Concentric (880) Rectangular [83:17]	ANN	*B, H, t, L, f*_*y*_*, Es, f*_*c*_’ (*P*_*u*_)	R^2^ = 0.9956, a20-index = 92.5%, RMSE = 154.66, MAPE% = 7.54, VAF = 99.118
Wang^26^	Eccentric (403) Rectangular [80:20]	SVR, RFR, ANN	*A*_*c*_*f*_*c*_’*, e/H, e, A*_*s*_*f*_*y*_*, H, EI*_*s*_*, B, λ*_*g*_*, f*_*c*_’*, L, t, EI*_*c*_*, N*_*pl*_ (*P*_*u*_)*	SVR: μ = 1.01, MAPE% = 5, R2 = 0.99, a20 = 96%, RMSE = 92,647 RFR: μ = 1.03, MAPE% = 9, R2 = 0.96, a20 = 91%, RMSE = 187,170 ANN: μ = 1.03, MAPE% = 12, R2 = 0.97, a20 = 86%, RMSE = 171,021
Memarzadeh^25^	Concentric (347) Square [85:15]	GEP, ANN	*f*_*y*_*, f*_*c*_’*,A*_*c*_*,A*_*s*_*, B/t,*$\lambda$ (*P*_*u*_)	**GEP**: R = 0.98, CoV = 0.23, RSE = 0.17, RMSE = 464 **ANN** R = 0.99, CoV = 0.12, RSE = 0.01, RMSE = 254
This study	Concentric (958) Eccentric (405) Rectangular [80:20]	GPR, XGB, SVR, ANN	*L, H, B, t, f*_*y*_*, f*_*c*_’*, e*_*t*_*, e*_*b*_ (*P*_*u*_*/N*_*pl*_)	GPR (concentric): μ = 0.998, CoV = 0.058, R^2^ = 0.996, MAPE = 3.78, a20 = 99 GPR (eccentric): μ = 1.003, CoV = 0.055, R^2^ = 0.996, MAPE = 3.41, a20 = 98.8 See Table 3 for remaining models

* *λ*_*g*_ is the global slenderness ratio, *EI*_*s*_*, EI*_*c*_*,* are the flexural stiffness of steel and concrete materials, respectively. *N*_*pl*_ is the sum of strength of steel and concrete material defined in Eq. (11).

The ML techniques mentioned earlier can be effectively combined with metaheuristic optimization methods^27^, such as particle swarm optimization (PSO)^28^ and grey wolf optimization (GWO)^29^. Metaheuristic optimization methods are specifically designed to mitigate the issue of getting trapped in local minima during optimization, unlike traditional optimization methods, such as gradient-based approaches. Several models have been employed in the literature for hybrid computational intelligence methods^22,23,30,31^. Ren et al.^22^ used a hybrid model based on an SVR, with parameters optimized using PSO to investigate the axial capacity of short square CFST columns.

Generally, ML can offer an innovative approach to predicting the capacity of CFST columns. Although various ML models have been introduced for CFST column predictions, as shown in Table 1, further work is necessary, primarily for the following reasons. First, most studies focused on predicting the loading capacity of RCFST columns under axial loads, with less exploration of their behavior under diverse loading conditions. Second, most studies focus on using ANN and SVR to predict the compression strength of CFST columns, and other ML algorithms, such as the Gaussian process (GPR) and the extreme gradient boosting (XGBoost) model^32^, are less commonly employed and require further exploration. Third, many researchers directly used axial strength as the output parameter despite its skewed and biased statistical distribution. In addition, the axial strength fails to capture the physical properties of CFST columns, such as the confinement efficiency of the CFST column and the effect of the local and global slenderness ratios. This paper introduces a dimensionless strength index as an alternative output parameter to address these limitations.

The primary objective of this research is to introduce several ML models, including the Gaussian process (GPR)^19^, extreme gradient boosting model (XGBoost)^32,33^, support vector regression^18^ optimized by the particle swarm optimization method (PSVR), and artificial neural network (ANN), for predicting the compressive resistance of RCFST columns under axial and eccentric loadings.

## Gaussian process model

**Figure 1:**
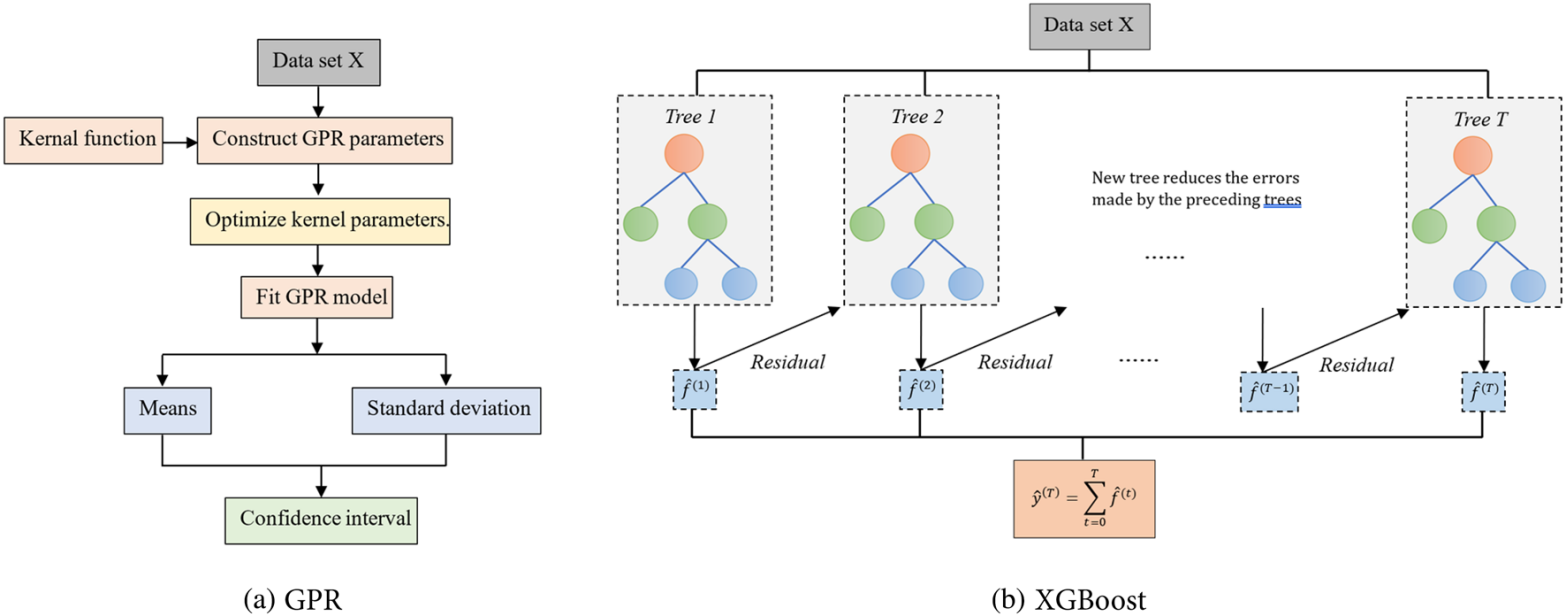
Flow charts of the introduced ML models.

Gaussian processes (GPR)^19^ are an ML method based on Bayesian and statistical learning theories. GPR defines a distribution over functions, as defined in Eq. (1), reasoning about functions based on observed data points. This technique can effectively handle uncertainty and adapt to noise and complexity levels.

1 $$f\left(x\right)\sim GP\left(m\left(x\right),K\left(x,{x}^{\mathrm{^{\prime}}}\right)\right),$$

where *f(x)* is the function value at input $x$, $m\left(x\right)$ is the prior mean function, and $K\left(x,{x}^{\mathrm{^{\prime}}}\right)$ is the covariance (kernel) function determining the covariance between any inputs x and ${x}^{\mathrm{^{\prime}}}$. A combination of kernels, including the Gaussian kernel, Matern kernel, and periodic kernel, are used together to capture different aspects of the data, such as the overall level, smoothness, noise, and variations. The kernel parameters are optimized by maximizing the log-marginal-likelihood^19^. The mean procedures of the GPR are introduced in Fig. 1(a). Given observed input‒output pairs, GPR allows predictions for new inputs by inferring a Gaussian distribution over functions as follows:

2 $$p\left(f\left(x\right)|X,y\right)\sim N\left(f\left(x\right)|{\mu }_{p}\left(X\right),{\Sigma }_{p}\left(X\right)\right),$$

where the posterior distribution $p\left(f\left(x\right)|X,y\right)$ is also a Gaussian distribution with a posterior mean function ${\mu }_{p}\left(X\right)$ and a posterior covariance function ${\Sigma }_{p}\left(X\right)$ defined, respectively, as follows:

3 $${\mu }_{p}\left(x\right)=m\left(x\right)+{K}^{T}\left(X,x\right){\left[K+{\sigma }_{n}^{2}I\right]}^{-1}\left(y-m\left(X\right)\right),$$

4 $${\Sigma }_{p}\left(x\right)=K\left(x,x\right)- {K}^{T}\left(X,x\right){\left[K+{\sigma }_{n}^{2}I\right]}^{-1}K\left(X,x\right),$$

where ${\mu }_{p}\left(x\right)$ and ${\Sigma }_{p}\left(x\right)$ define the mean prediction of the new input point x and the uncertainty (variance) associated with each prediction. The mean procedures of the GPR are introduced in Fig. 1a.

## Extreme gradient boosting model

The extreme gradient boosting (XGBoost)^32^ model builds upon the foundation of gradient boosting trees (GBDTs) by introducing algorithmic enhancements, including robustness, effectiveness, and scalability for large-scale datasets. XGBoost uses an ensemble of decision trees as its base learners. These decision trees are often shallow typically called weak learners. Combining multiple simple trees helps reduce overfitting and improves model generalization. XGBoost aims to reduce the sum of two key components: the training error and regularization, as illustrated in Eq. (5).

5 $$ob{j}^{t}=\sum_{i=1}^{n} L\left({y}_{i}\right)+\Omega \left({f}_{t}\right),$$

where *L* represents the loss function, quantifying the difference between the predicted and the actual value, and Ω denotes the regularization term, controlling the model complexity to prevent overfitting. The second-order Taylor approximation of the loss function can be written in Eqs. (6–8).

6 $$ob{j}^{t}=\sum_{i=1}^{n} L\left({y}_{i},{\widehat{y}}_{i}^{t}\right)+\sum_{i=1}^{t}\Omega \left({f}_{i}\right)=\sum_{i=1}^{n} L\left({y}_{i},{\widehat{y}}_{i}^{(t+1)}+{f}_{t}\left({x}_{i}\right)\right)+\Omega \left({f}_{t}\right)+\text{ constant}$$

7 $$ob{j}^{t}=\sum_{i=1}^{n} \left[L\left({y}_{i},{\widehat{y}}_{i}^{(t-1)}\right)+{g}_{i}{f}_{t}\left({x}_{i}\right)+\frac{1}{2}{h}_{i}{f}_{t}^{2}\left({x}_{i}\right)\right]+\Omega \left({f}_{t}\right)+\text{ constant}$$

8 $${g}_{i}={\partial }_{{\widehat{y}}_{i}\left(t-1\right)} L\left({y}_{i},{\widehat{y}}_{i}^{\left(t-1\right)}\right), \quad {h}_{i}={\partial }_{{\widehat{y}}_{i}^{\left(t-1\right)}}^{2} L\left({y}_{i},{\widehat{y}}_{i}^{\left(t-1\right)}\right)$$

The fundamental tree employed in this study is a simple regression tree, defined by Eq. (9).

9 $$\Omega \left({f}_{t}\right)=\gamma T+\frac{1}{2}\lambda \sum_{j=1}^{T} {\omega }_{j}^{2} ,$$

where *γ* represents the penalty factor, *T* defines the count of leaf nodes, and *ω*_*j*_ defines the weighting assigned to the leaf *j*. Disregarding the constant term, the objective function reduces to the form in Eq. (10).

10 $$obj=\sum_{j=1}^{T} \left[\left(\sum {g}_{i}\right){\omega }_{j}+\frac{1}{2}\left(\sum {h}_{i}+\lambda \right){\omega }_{j}^{2}\right]+\gamma T$$

The superiority of XGBoost over other ensemble techniques can be attributed to its mechanism of integrating several weaker base learners to form a stronger model through a process known as boosting. Boosting is an iterative training process such that training a new decision tree requires reducing the errors made by the preceding trees in prior iterations. The flow chart of the XGBoost model is illustrated in Fig. 1b.

## Database description

To construct a precise model for predicting the strength of RCFST columns, a comprehensive experimental database was compiled, consisting of 958 tests conducted on RCFST columns subjected to axial loading (Database 1) and 405 tests on RCFST columns subjected to eccentric loading (Database 2)^3–5^. While these experimental tests may not be identical in terms of their testing conditions, they are substantial in volume and diverse in sources, simulating different real-world manufacturing scenarios. RCFST columns subjected to monotonic axial loading are selected, where the entire cross-sections, i.e., concrete and steel tube, are fully loaded. Only CFST columns with normal and high-strength concrete and low-carbon steel tubes are collected. Specimens with stainless steel tubes, aluminum tubes, recycled aggregate concrete, steel fiber concrete, etc., are excluded.

**Figure 2:**
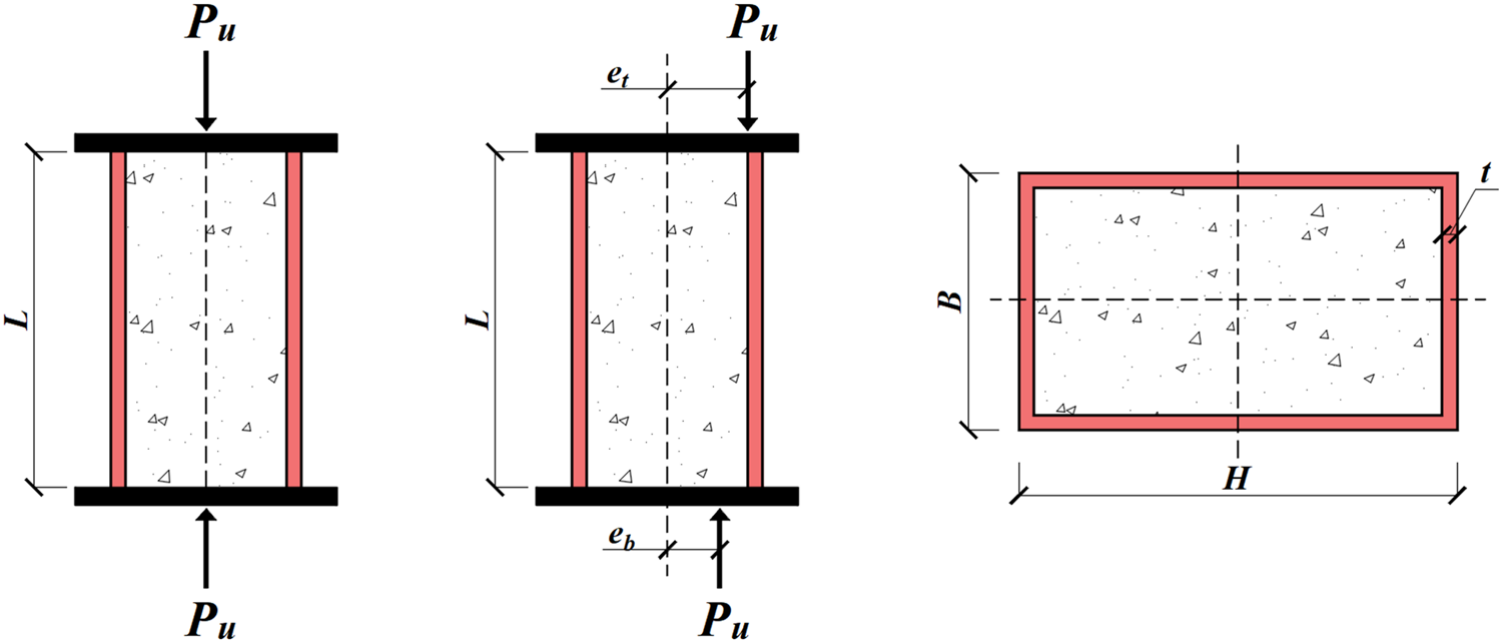
RCFST column configurations under axial and eccentric loading conditions.

**Figure 3:**
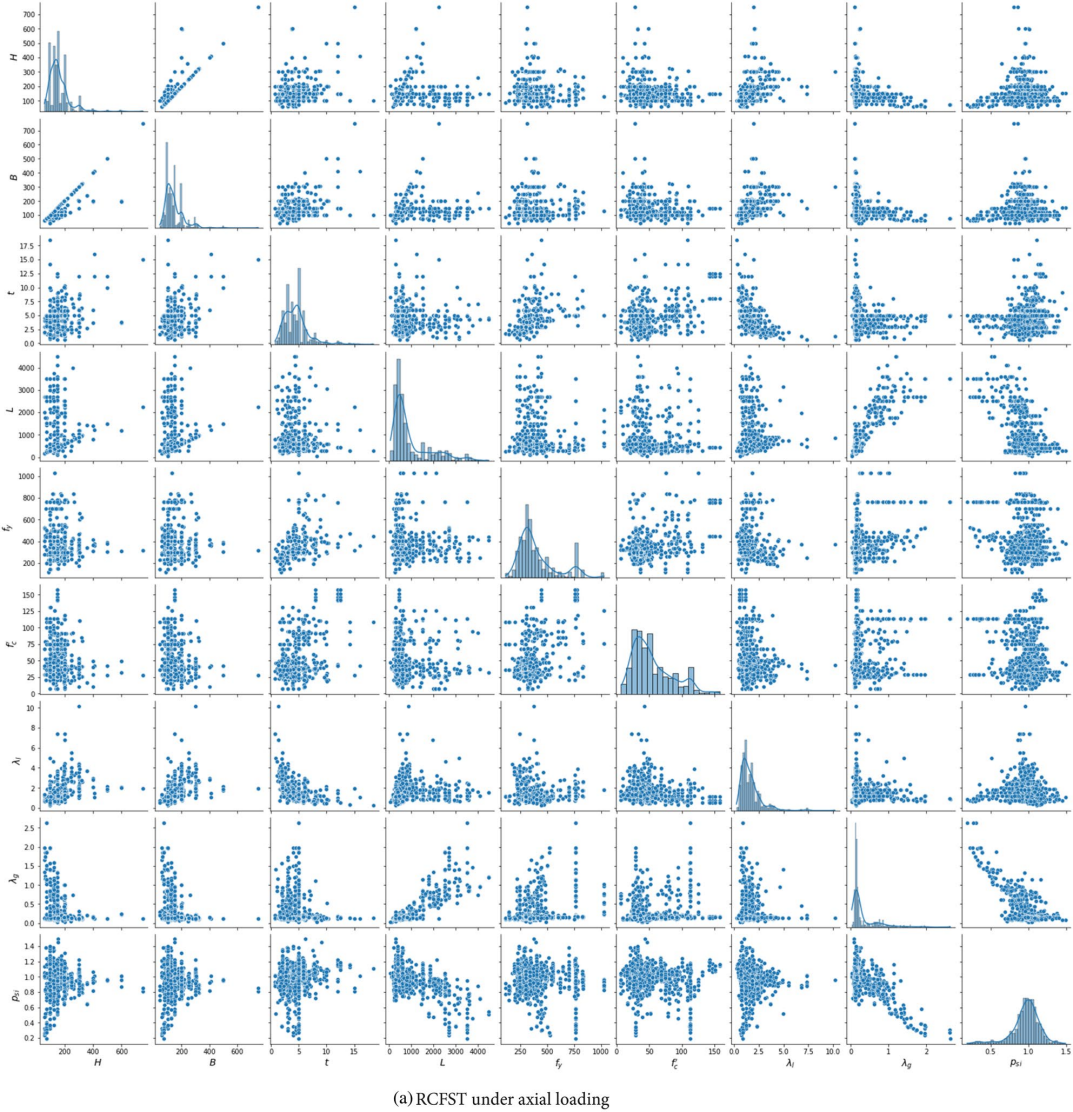

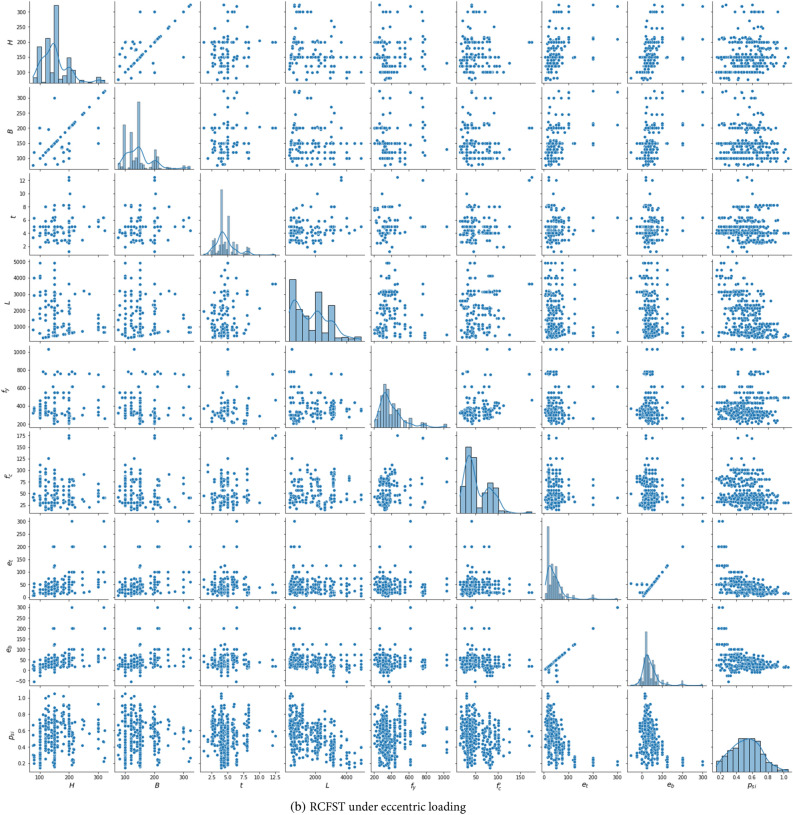
Distribution of the two databases.

As illustrated in Fig. 2, the input variables include geometric variables, including the column width (*B*), column height (*H*), steel tube thickness (*t*), column length (*L*), load top eccentricity (*e*_*t*_), and load bottom eccentricity (*e*_*b*_), as well as material properties, including steel yield strength (*f*_*y*_) and concrete compressive strength (*f*_*c*_’). Naser et al.^21^ suggested that the remaining material properties of concrete and steel, i.e., Young’s modulus of steel (*E*_*s*_) and concrete (*E*_*c*_) and the ultimate strength of steel (*f*_*u*_), have no significant influence on the training of data-driven models. The statistical distributions of these databases are presented in Fig. 3 and Table 2.

**Table 2:** Statistic features of the experimental dataset.

Column type	Variable	Symbol	Type	Statistics					
				Min	Max	Mean	Std	Skewness	Kurtosis
Database 1	Height of outer tube	H (mm)	Input	60	750	160.9	74.31	2.89	14.25
	Width of the outer tube	B (mm)	Input	44	750	144.9	68.15	2.85	15.75
	Thickness of the outer tube	$t$ (mm)	Input	0.7	18.5	4.44	2.21	1.71	5.27
	Column length	$L$ (mm)	Input	60	4500	951.9	854.2	1.66	1.99
	Yield strength of outer tube	${f}_{y}$ (MPa)	Input	115	1031	398.2	174.2	1.43	1.51
	Concrete strength	${f}_{c}{\prime}$ (MPa)	Input	7.02	157.5	54.67	30.63	0.96	0.28
	Local slenderness ratio	*λ*_*l*_	–	0.25	10.16	1.633	1.008	2.62	11.76
	Global slenderness ratio	*λ*_*g*_	–	0.024	2.64	0.317	0.367	2.72	8.78
	Axial load	${P}_{u}$ (MPa)	–	105	24,294	2297	2266	3.75	23.03
	Strength index	${p}_{si}$	Output	0.19	1.493	0.97	0.176	− 1.2	3.25
Database 2	Height of outer tube	H (mm)	Input	76.2	323	152.8	47.31	1.3	2.24
	Width of the outer tube	B (mm)	Input	76.2	323	147.5	46.12	1.34	2.41
	Thickness of the outer tube	$t$ (mm)	Input	1.25	12.5	4.63	1.59	1.41	3.47
	Column length	$L$ (mm)	Input	330	4910	1753	1107	0.6	− 0.44
	Yield strength of outer tube	${f}_{y}$(MPa)	Input	205	1031	396.8	140.9	2.05	5.42
	Concrete strength	${f}_{c}{\prime}$ (MPa)	Input	15	175.9	53.7	27.91	1.02	1.04
	Top-end eccentricity	${e}_{t}$ (mm)	Input	6	300	43	37	3.36	15.64
	Bottom-end eccentricity	${e}_{b}$ (mm)	Input	− 55	300	40.06	39.26	2.86	12.84
	Axial load	${P}_{u}$ (MPa)	–	156	7136	1187	945.7	2.36	8.25
	Strength index	${p}_{si}$	Output	0.15	1.053	0.52	0.183	0.14	− 0.41

**Figure 4:**
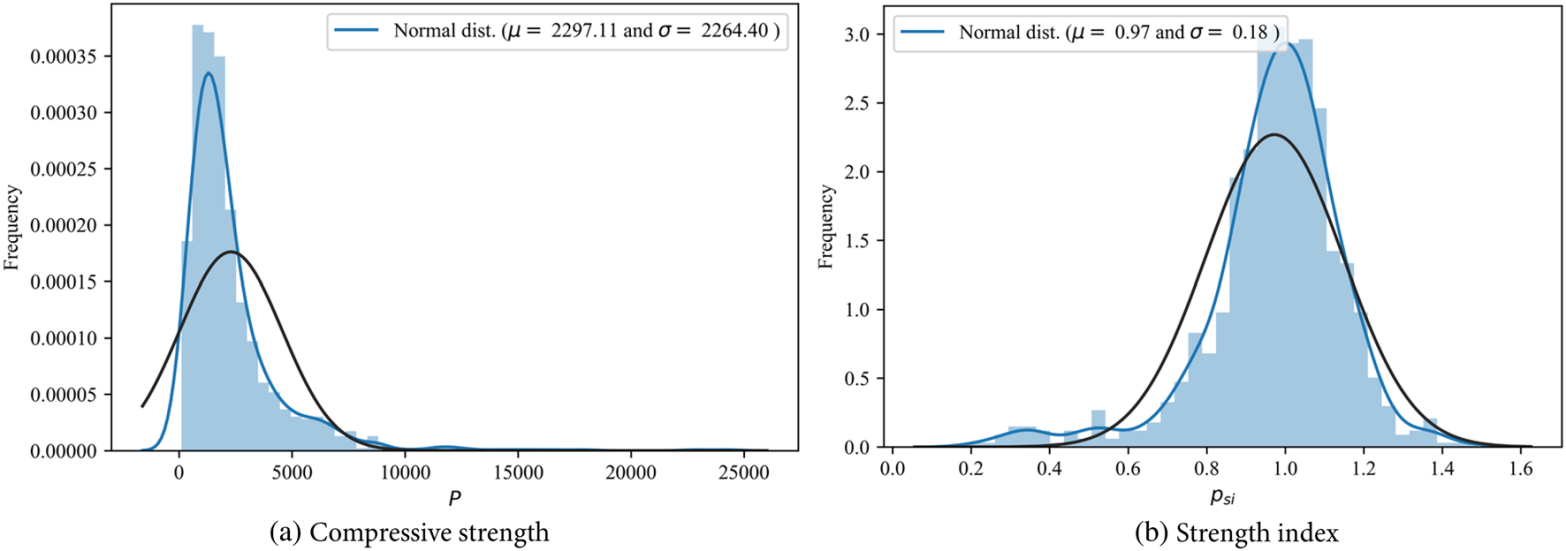
Frequency histogram of compressive strength and strength index for database 1.

Generally, ML models perform better when working with data that follow a roughly normal distribution. However, the axial capacity distribution for the RCFST columns shown in Fig. 4a exhibits significant skewness, which can negatively impact model performance. A dimensionless strength index, denoted as *p*_*si*_, is introduced as the main output parameter to address this issue. It is defined by dividing the column axial load by the sum of the individual strengths of its components, as given in Eq. (11).

11 $${p}_{si}=\frac{{P}_{u}}{{N}_{pl}}, {N}_{pl}={A}_{s}{f}_{y}+{A}_{c}{f}_{c}{\prime},$$

where *A*_*s*_ and *A*_*c*_ are the outer steel tube and concrete areas, respectively. This introduced index can reflect the confinement efficiency of the CFST column, i.e., a relatively high value of the strength index indicates high confinement exerted by the outer tube. Furthermore, the statistical distribution of the strength index resembles a normal distribution, as shown in Fig. 4b and Table 2, enhancing predictability performance.

**Figure 5:**
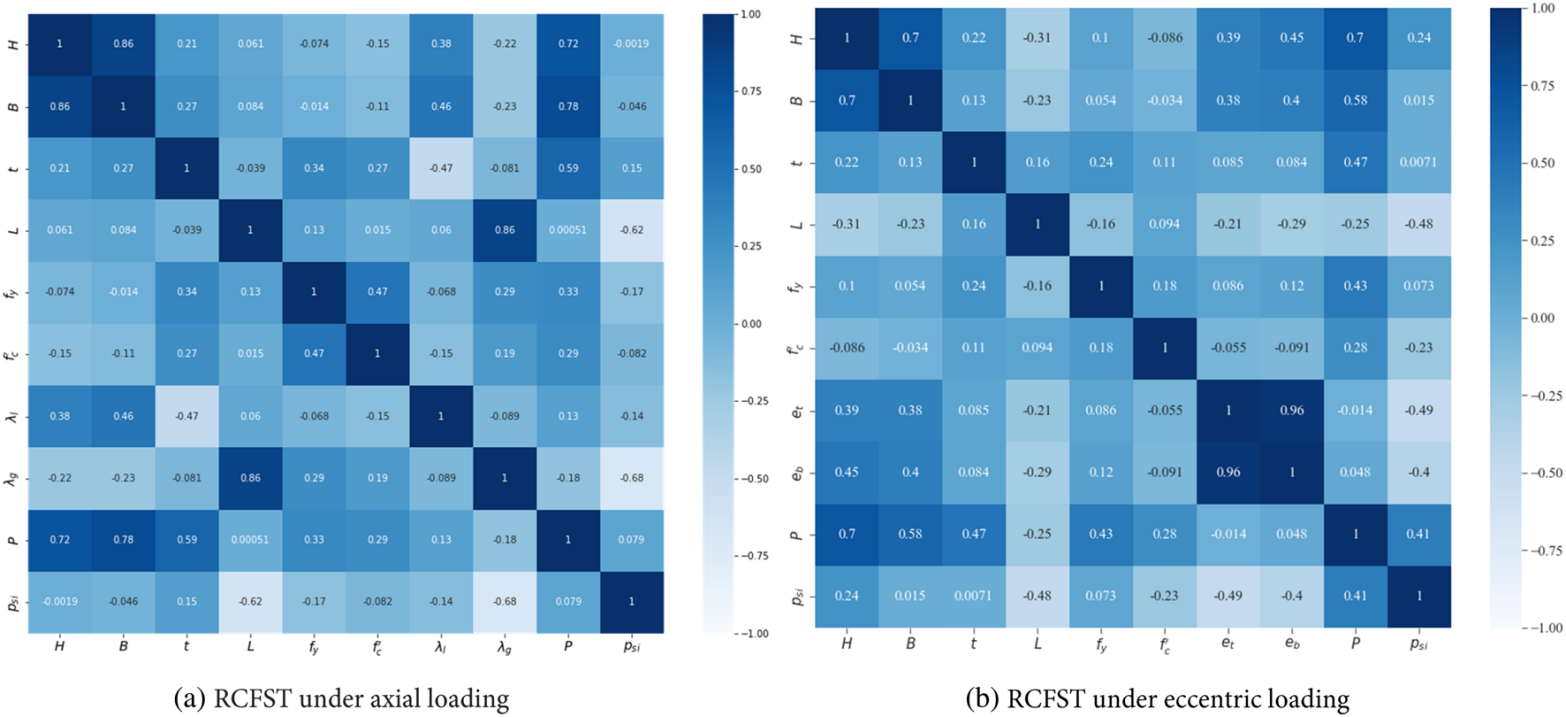
Correlation matrix for the RCFST columns databases under axial and eccentric loading conditions.

Additionally, the correlations between all input and output variables in the databases are investigated through the Pearson correlation coefficient and are displayed in Fig. 5. There is a relatively strong correlation between the input variables and the axial capacity (*P*) across different datasets, negatively impacting the predictivity performance. However, the correlations between the input variables and the strength index (*p*_*si*_) are less significant. In addition, as shown in Figs. 3 and 5, increasing the load eccentricity, global slenderness* λ*_*g*_, or local slenderness *λ*_*l*_, defined in Eq. (12), reduces the column strength index. These observations align well with the experimental behavior of CFST columns. These findings indicate the benefits of using the strength index as an output variable instead of the axial capacity.

12 $${\lambda }_{l}=\frac{H}{t}\sqrt{\frac{{f}_{y}}{{E}_{s}}}, {\lambda }_{g}=\sqrt{\frac{{N}_{p}}{{N}_{cr}}}, {N}_{p}={f}_{y}{A}_{s}+0.85{f}_{c}{\prime}{A}_{c},{N}_{cr}=\frac{{\pi }^{2}\left(E{I}_{eff}\right)}{{L}^{2}},E{I}_{eff}={E}_{s}{I}_{s}+0.6{E}_{c}{I}_{c}$$

where* E*_*s*_*I*_*s*_ and *E*_*c*_*I*_*c*_ are the flexural stiffness of steel and concrete materials.

It is important to acknowledge that the parameter ranges of CFST samples in the databases fall outside the scope of existing design codes^10,11^, as illustrated in Table 2 and Fig. 3. This aspect can be advantageous in training machine learning models with broader applicable ranges. In addition, the axial database covers a wide range of steel section slenderness, including both compact, noncompact, and slender sections (*λ*_*l*_ coefficient ranges from 0.25 up to 10.17)^11^. In addition, a wide range of global slenderness is covered, ranging from 0.0243 to 2.64, covering short (*λ*_*g*_ < 0.5 as recommended by Eurocode 4^10^) and long columns. Furthermore, the database encompasses a wide range of concrete and steel strengths. The introduced databases include both traditional materials (with *f*_*c*_*’* values below 70 MPa and *f*_*y*_ values below 460 MPa, as suggested by AISC 360-22^11^) and higher strength classes (with *f*_*c*_*’* up to 175.9 MPa and *f*_*y*_ up to 1031 MPa). While a wide range of material strengths is considered, their distributions are not uniform. Specifically, steel strength tends to cluster in the 200–800 MPa range, with only a limited number of samples exceeding 800 MPa. In the case of concrete strength, most specimens fall within the 20–100 MPa range, with a smaller subset exceeding 100 MPa. ML models rely on the information contained in the input data. However, the scarcity of training data within a specific range of an input feature can lead to insufficient learning for that range. Consequently, the application of the trained machine learning model might encounter challenges when applied to data falling within a range for which the model lacks sufficient training.

## Performance and results of ML models

**Figure 6:**
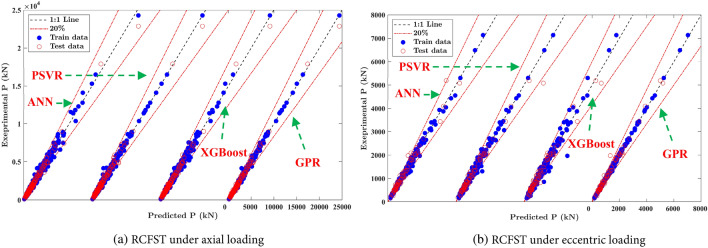
Comparison between ML models for training and testing datasets.

Data normalization is performed using the min–max scaling technique to mitigate the impact of multidimensionality and ensure numerical stability. During the training phase, the grid searching technique was employed for tuning the model hyperparameters, and fivefold cross-validation was utilized to reduce overfitting issues. As recommended by Nguyen 2020^23^ and other studies^24,26^, eighty percent of the original dataset was chosen randomly for training, leaving the remaining 20% to test the models. To compare and evaluate the effectiveness and reliability of the introduced models, two different ML models, including the support vector machine integrated with particle swarm optimized (PSVR)^22^ and ANN models, were introduced. Figure 6 illustrates the relation between the predictions generated by the four ML models and the experimental results. It is evident from Fig. 6 that the scatter between the predicted and experimental results for the four ML models closely follows the diagonal line, falling mostly within the ± 20% margins for the training and test subsets. Table 3 presents the evaluation metrics to assess the prediction accuracy for these ML models: the mean (μ), coefficient of variation (CoV), coefficient of determination (R^2^), root mean squared error (RMSE), mean absolute percentage error (MAPE), a20-index, Nash–Sutcliffe efficiency (NSE), Willmott index of agreement (d), and confidence index (CI)^34^. These measures are defined as:

**Table 3:** Comparison of the developed ML models for different databases.

Metrics*	Training data				Testing data				All data					
	GPR	XGB	PSVR	ANN	GPR	XGB	PSVR	ANN	GPR	XGB	PSVR	ANN	EC4	AISC
Database 1														
Mean μ	0.998	0.998	0.999	0.999	0.999	1.002	0.996	0.999	0.998	0.999	0.999	0.999	1.12	1.16
CoV	0.049	0.072	0.083	0.093	0.086	0.087	0.099	0.102	0.058	0.075	0.086	0.095	0.13	0.16
R^2^	0.997	0.992	0.995	0.989	0.993	0.993	0.991	0.987	0.996	0.992	0.994	0.988	0.96	0.94
MAPE%	3.226	5.061	4.923	7.125	5.992	6.217	6.861	7.815	3.78	5.292	5.311	7.264	14.4	18.7
RMSE	0.043	0.064	0.08	0.09	0.08	0.079	0.092	0.095	0.052	0.068	0.083	0.091	0.15	0.18
a20-index	0.996	0.98	0.965	0.96	0.964	0.969	0.948	0.938	0.99	0.978	0.961	0.955	0.74	0.64
NSE	0.968	0.925	0.885	0.849	0.872	0.871	0.825	0.805	0.951	0.915	0.874	0.842	0.8	0.76
d	0.957	0.918	0.88	0.868	0.877	0.882	0.851	0.848	0.938	0.911	0.874	0.864	0.73	0.67
CI	0.926	0.849	0.779	0.737	0.765	0.768	0.702	0.683	0.892	0.834	0.764	0.727	0.58	0.51
Database 2														
Mean μ	0.999	0.987	0.996	0.998	1.018	1.027	1.022	1.015	1.003	0.995	1.001	1.002	1.07	1.07
CoV	0.035	0.106	0.098	0.069	0.098	0.128	0.106	0.106	0.055	0.113	0.1	0.078	0.14	0.22
R^2^	0.999	0.978	0.984	0.993	0.985	0.973	0.985	0.969	0.996	0.977	0.984	0.989	0.96	0.91
MAPE%	2.536	7.552	6.773	5.376	6.888	9.765	8.387	7.94	3.406	7.995	7.096	5.889	12.4	14.7
RMSE	0.018	0.049	0.052	0.036	0.05	0.068	0.06	0.054	0.028	0.053	0.054	0.04	0.08	0.09
a20-index	1.0	0.92	0.938	0.988	0.938	0.877	0.901	0.926	0.988	0.911	0.931	0.975	0.79	0.78
NSE	0.995	0.961	0.958	0.98	0.958	0.916	0.935	0.95	0.988	0.952	0.954	0.975	0.89	0.87
d	0.991	0.953	0.944	0.97	0.944	0.912	0.927	0.937	0.981	0.944	0.940	0.963	0.89	0.87
CI	0.986	0.916	0.904	0.951	0.904	0.835	0.867	0.89	0.969	0.899	0.897	0.939	0.79	0.76

$$\mu =\frac{1}{n}\sum_{i=1}^{n}\frac{{\widehat{y}}_{i}}{{y}_{i}}, {R}^{2}=1-\frac{\sum_{i=1}^{n}{\left({\widehat{y}}_{i}-{y}_{i}\right)}^{2}}{\sum_{i=1}^{n}{\left({\widehat{y}}_{i}-\overline{y }\right)}^{2}}, RMSE=\sqrt{\frac{1}{n}\sum_{i=1}^{n}{\left({\widehat{y}}_{i}-{y}_{i}\right)}^{2}}, MAPE=\frac{1}{n}\sum_{i=1}^{n}\left|\frac{{\widehat{y}}_{i}}{{y}_{i}}-1\right|\times 100\%,$$

13 $$d=1-\frac{\sum_{i=1}^{n}{\left({\widehat{y}}_{i}-{y}_{i}\right)}^{2}}{\sum_{i=1}^{n}{\left(\left|{\widehat{y}}_{i}-\overline{y }\right|+\left|{y}_{i}-\overline{y }\right|\right)}^{2}}, NSE=1-\frac{\sum_{i=1}^{n}{\left({\widehat{y}}_{i}-{y}_{i}\right)}^{2}}{\sum_{i=1}^{n}{\left({y}_{i}-\overline{y }\right)}^{2}}, CI=NSE\times d$$

where ${y}_{i}$ defines the actual output value of the *i-*th sample, ${\widehat{y}}_{i}$ is the output value of the *i*-th sample, $\overline{y }$ is the mean value of experimental observations, and *n* is the number of specimens in the database. The a20-index^15,35^ is a percentile-based metric that measures the partition of samples for which the absolute differences between predicted and observed results exceed 20%.

As observed in Table 3, the prediction accuracy of the introduced ML models exhibits little difference with R^2^ and mean values approaching 1.0 and CoV values less than 0.113. The predictions of all proposed models have error values lower than 20% for 95.5% of axially loaded specimens and 91.1% of the eccentrically loaded specimens. Similar performance can be found for the remaining metrics. Table 3 reveals that the GPR model introduces the best evaluation metrics for the training and testing subsets, with MAPE% values equal to 3.78% and 3.41% for the axially and eccentrically loaded column datasets, respectively, followed in accuracy by the XGBoost model for the axially loaded column dataset and the ANN model for the eccentrically loaded column dataset. The ANN and XGBoost models are the least accurate for axially and eccentrically loaded column datasets, respectively, while the PSVR model introduces moderate prediction accuracy. In addition, the evolution metrics of the testing sets exhibit similar results to the training sets, indicating minimizing the overfitting issues.

The predictions by the introduced models were compared with the existing codes in Table 3, including Eurocode 4 (EC4)^10^ and AISC360^11^. The mean values of code methods are all above 1.0, representing conservative predictions. This result is reasonable as design codes are inclined to be conservative to yield safer designs. In addition, the accuracy of the introduced ML models is significantly higher than that of the two design standards, particularly noticed when evaluating a20-index. For instance, 99%, 97.8%, 96.1%, and 95.5% of the concentrically loaded CFST database obtained, respectively, from GPR, XGBoost, PSVR, and ANN models exhibit error rates within 20%, much higher than the 74% and 64% proportions reported by EC4^10^ and AISC 360-22^11^, respectively. Furthermore, the RMSE and MAPE of EC4 and AISC360^11^ predictions are approximately two to four times those of ML models, indicating the better performance of ML models compared to available standards. These findings can be attributed to the fact that AISC 360-22 neglects the confinement interaction between steel and concrete materials, and EC4 disregards the local buckling effect and imposes a limitation for the slenderness ratio *λ*_*l*_.

Compared with some ML models introduced in the literature, as summarized in Table 1, the developed models achieved notable improvement in prediction accuracy. The introduced GPR model exhibits an a20-index of 98.8%, surpassing the models introduced by Wang et al.^26^ (a20-index = 96%) and the GPR model proposed by Le et al.^15^ (a20-index = 92.5%). The enhanced performance of the introduced GPR model compared to the GPR model of Le et al.^15^ can be attributed to using a combination of kernels, which can capture various aspects of the data, including smoothness, noise, and variations. Furthermore, the MAPE of the proposed GPR model stands at 3.41, which is considerably lower than that of the SVR models proposed by Ren et al.^22^ and Nguyen et al.^24^.

**Figure 7:**
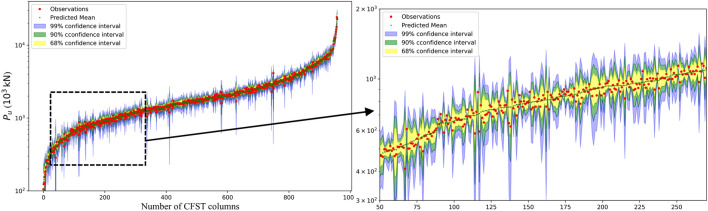
Gaussian process regression on a semilog scale on the y-axis for axially loaded column database.

In addition to the relatively high accuracy of the GPR model, it can provide the confidence intervals for the prediction results, as shown in Fig. 7 for the axially loaded column database. This quantification of uncertainty enhances its applicability in guiding practical design considerations. The even distribution of the predicted column strength around the measured strength, as depicted in Fig. 7, further confirms the accurate predictive capabilities of the GPR model for RCFST column strength.

## Feature importance analysis

**Figure 8:**
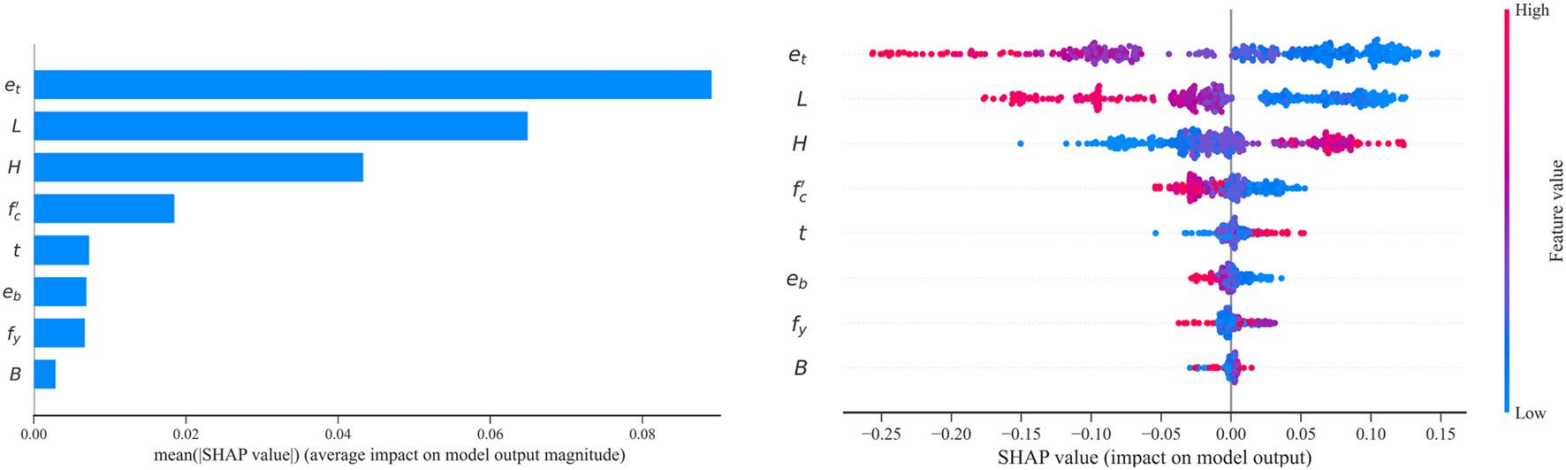
Summary plot and SHAP feature importance for the eccentrically loaded RCFST column database.

Analyzing the impact of input parameters on compressive strength is a crucial guiding factor in designing RCFST columns. In this study, the Shapley Additive Explanation (SHAP) method is utilized to assess the impact of input parameters on the strength index^33,36^. As depicted in Fig. 8, a feature value larger than zero signifies a positive correlation between the variable and the strength index. In contrast, a feature value less than zero indicates a negative impact on the strength index. For RCFST columns under eccentric loading, the top-end eccentricity (*e*_*t*_) and column length (*L*) emerge as the most influential design parameters within the collected database. The feature importance of the remaining variables is ranked from highest to lowest. Furthermore, it can be deduced that, except for column width (B), height (H), and steel tube thickness (t), all remaining input variables have a negative influence on the strength index, indicating that an increase in these parameters reduces the strength index. Increasing column height and steel thickness enhance the flexural strength and confinement behavior of RCFST columns while increasing column length and load eccentricity reduce the column capacity strength. These findings agree well with the experimental results.

## Limitations and future works

This section outlines the limitations of the established data-driven models and highlights potential areas for future research. The validity of the proposed model is constrained within the range of minimum and maximum values for each input parameter, as outlined in Table 2. These values not only define the applicability of the computational model but also set the boundaries within which accurate predictions can be made. In addition, considering the uneven distribution of certain parameters, as explained in Fig. 3, applying the ML models needs caution where the input features fall within ranges lacking sufficient training data, and experimental studies are needed to enrich the database within these less-represented ranges.

An innovative methodology that can be considered involves integrating finite element modeling with the GPR model within the *Design of Experiments* (DOEs) framework. This approach is designed to identify and select the optimal training points that can effectively reduce errors through adaptive learning and use the predictive capabilities of finite element modeling to model these critical points. The accuracy of predictive models can be substantially enhanced, yielding more efficient and reliable ML models for CFST columns.

## Conclusions

This study introduces two ML models, including the Gaussian process (GPR) and extreme gradient boosting (XGBoost) models, for predicting the compressive resistance of rectangular concrete-filled steel tubular (CCFST) columns subjected to axial and eccentric loading conditions. These models are compared with other ML models, including support vector regression optimized by particle swarm optimization (PSVR), an artificial neural network (ANN), and previous ML studies. The key findings are summarized as follows:

1. The provided ML models can effectively capture the complicated relationship between geometric and material parameters and compressive resistance for RCFST columns subjected to different loading conditions.
2. The proposed normalization approach of the axial load by introducing the strength index yields a nearly normal distribution, which improves model performance and robustness. In addition, using the strength index as an output parameter reflects insights into the level of strength in terms of local and global buckling.
3. The GPR model is the most accurate and reliable model, with MAPE% less than 4%. In addition, the remaining ML models offer acceptable accuracy with MAPE% less than 8%. This high prediction accuracy promotes using the ML techniques as valuable tools alongside design code standards for estimating the compressive strength of RCFST columns.
4. Compared with existing standards and ML studies, the developed models achieved better performance in prediction accuracy. The predictions of all proposed models have error values lower than 20% for 95.5% of axially loaded specimens and 91.1% of the eccentrically loaded specimens, much higher than the proportions reported by EC 4. and AISC 360-22.
5. From feature importance analysis, top-end eccentricity and column length have the most negative influence on the strength index of RCFST columns. Therefore, designers should consider these parameters in optimizing and designing RCFST columns.

In summary, the proposed data-driven models can extract the axial compression capacity of RCFST columns with reliable and accurate results, making them valuable tools for structural engineers. While this paper illustrates the capability and precision of the introduced ML models for RCFST compressive strength prediction, future studies are needed to address the existing gaps in databases and to integrate the predictive capabilities of finite element modeling with ML models.

### Research significance

This study introduces two machine learning (ML) algorithms for predicting the compressive strength of rectangular concrete-filled steel tubular (RCFST) columns under different loading conditions. It employs two powerful ML models, the Gaussian process (GPR) and the extreme gradient boosting (XGBoost) model. The employed techniques can be considered valuable tools alongside the design code standards and finite element analysis.

## Data Availability

The datasets used and/or analyzed during the current study are available from the corresponding author on reasonable request.

## References

[1] Xiong MX, Xiong DX, Liew JYR (2017). Axial performance of short concrete filled steel tubes with high- and ultra-high- strength materials. Eng. Struct..

[2] Han LH, Li W, Bjorhovde R (2014). Developments and advanced applications of concrete-filled steel tubular (CFST) structures: Members. J. Constr. Steel Res..

[3] Thai SJ, Thai HT, Ngo T, Uy B, Kang WH, Hicks S (2020). Concrete-filled steel tubular (CFST) columns database with 3,208 tests. Mendeley Data.

[4] Goode CD (2008). Composite columns-1819 tests on concrete-filled steel tube columns compared with Eurocode 4. Struct. Eng..

[5] Denavit, M. D. *Characterization of behavior of steel-concrete composite members and frames with applications for design*. (University of Illinois at Urbana-Champaign, 2012).

[6] Tao Z, Han LH, Zhao XL (2004). Behaviour of concrete-filled double skin (CHS inner and CHS outer) steel tubular stub columns and beam-columns. J. Constr. Steel Res..

[7] Ho JCM, Lam JYK, Kwan AKH (2010). Effectiveness of adding confinement for ductility improvement of high-strength concrete columns. Eng. Struct..

[8] Du Y, Chen Z, Richard Liew JY, Xiong M-X (2017). Rectangular concrete-filled steel tubular beam-columns using high-strength steel: Experiments and design. J. Constr. Steel Res..

[9] Tao Z, Bin Wang Z, Yu Q (2013). Finite element modelling of concrete-filled steel stub columns under axial compression. J. Constr. Steel Res..

[10] BEng, S. H. & Park, S. EN 1994-eurocode 4: Design of composite steel and concrete structures. *Retrieved May*, vol. 10, p. 2022 (1994).

[11] AISC. AISC 360-22 specification for structural steel buildings. *Am. Inst. Steel Constr.*, p. 780. (2022).

[12] Ahmadi M, Naderpour H, Kheyroddin A (2014). Utilization of artificial neural networks to prediction of the capacity of CCFT short columns subject to short term axial load. Arch. Civ. Mech. Eng..

[13] Ahmadi M, Naderpour H, Kheyroddin A (2017). ANN model for predicting the compressive strength of circular steel-confined concrete. Int. J. Civ. Eng..

[14] Du Y, Chen Z, Zhang C, Cao X (2017). Research on axial bearing capacity of rectangular concrete-filled steel tubular columns based on artificial neural networks. Front. Comput. Sci..

[15] Le T-T, Asteris PG, Lemonis ME (2022). Prediction of axial load capacity of rectangular concrete-filled steel tube columns using machine learning techniques. Eng. Comput..

[16] Tran V-L, Thai D-K, Kim S-E (2019). Application of ANN in predicting ACC of SCFST column. Compos. Struct..

[17] Zarringol M, Thai H-T, Thai S, Patel V (2020). Application of ANN to the design of CFST columns. Structures.

[18] Suykens JAK, Vandewalle J (1999). Least squares support vector machine classifiers. Neural Process. Lett..

[19] Rasmussen, C. E. Williams, C. K. I. & others. in *Gaussian Processes for Machine Learning*, vol. 1. (Springer, 2006).

[20] Le T-T (2022). Practical machine learning-based prediction model for axial capacity of square CFST columns. Mech. Adv. Mater. Struct..

[21] Naser MZ, Thai S, Thai H-T (2021). Evaluating structural response of concrete-filled steel tubular columns through machine learning. J. Build. Eng..

[22] Ren Q, Li M, Zhang M, Shen Y, Si W (2019). Prediction of ultimate axial capacity of square concrete-filled steel tubular short columns using a hybrid intelligent algorithm. Appl. Sci..

[23] Nguyen, H. Q., Ly, H., Tran, V. Q. & Nguyen, T. Optimization of Artificial Intelligence System by Evolutionary Algorithm for Prediction of Axial Capacity of Rectangular Concrete Filled Steel Tubes under Compression. (2020). 10.3390/ma13051205 PMC7085075 32156033

[24] Nguyen MST, Kim SE (2021). A hybrid machine learning approach in prediction and uncertainty quantification of ultimate compressive strength of RCFST columns. Constr. Build. Mater..

[25] Memarzadeh A, Sabetifar H, Nematzadeh M (2023). A comprehensive and reliable investigation of axial capacity of Sy-CFST columns using machine learning-based models. Eng. Struct..

[26] Wang C, Chan TM (2023). Machine learning (ML) based models for predicting the ultimate strength of rectangular concrete-filled steel tube (CFST) columns under eccentric loading. Eng. Struct..

[27] Bianchi L, Dorigo M, Gambardella LM, Gutjahr WJ (2009). A survey on metaheuristics for stochastic combinatorial optimization. Nat. Comput. An Int. J..

[28] Kennedy, J. & Eberhart, R. Particle swarm optimization. In *Proceedings of ICNN’95*—*International Conference on Neural Networks*, vol. 4, pp. 1942–1948 (1995). 10.1109/ICNN.1995.488968

[29] Mirjalili S, Mirjalili SM, Lewis A (2014). Grey wolf optimizer. Adv. Eng. Softw..

[30] Yaseen ZM, Tran MT, Kim S, Bakhshpoori T, Deo RC (2018). Shear strength prediction of steel fiber reinforced concrete beam using hybrid intelligence models: A new approach. Eng. Struct..

[31] Ngo N-T, Le HA, Pham T-P-T (2021). Integration of support vector regression and grey wolf optimization for estimating the ultimate bearing capacity in concrete-filled steel tube columns. Neural Comput. Appl..

[32] Chen, T. & Guestrin, C. XGBoost: A scalable tree boosting system. In *Proceedings of the 22nd ACM SIGKDD International Conference on Knowledge Discovery and Data Mining*, pp. 785–794. (2016) 10.1145/2939672.2939785

[33] Wang J, Lu R, Cheng M (2023). Application of ensemble model in capacity prediction of the CCFST columns under axial and eccentric loading. Sci. Rep..

[34] El M (2021). Modeling the nonlinear behavior of ACC for SCFST columns using experimental-data and a novel evolutionary-algorithm. Structures.

[35] Asteris PG, Mokos VG (2020). Concrete compressive strength using artificial neural networks. Neural Comput. Appl..

[36] Abdallah MH (2023). The machine-learning-based prediction of the punching shear capacity of reinforced concrete flat slabs: An advanced M5P model tree approach. Appl. Sci..

